# Inflammatory Bowel Diseases and Nephropathies: Exploring the Gut–Kidney Axis

**DOI:** 10.3390/life14121541

**Published:** 2024-11-25

**Authors:** Roberto de Sire, Alessia La Mantia, Livio Bonacci, Anna Testa, Alessia Dalila Guarino, Antonio Rispo, Olga Maria Nardone, Fabiana Castiglione

**Affiliations:** 1 IBD Unit, Department of Clinical Medicine and Surgery, University Federico II, 80126 Naples, Italy; alessialamantia7@gmail.com (A.L.M.); liviobonacci@gmail.com (L.B.); annatesta82@virgilio.it (A.T.); ale.tizi@hotmail.it (A.D.G.); antonio.rispo2@unina.it (A.R.); olga.nardone@libero.it (O.M.N.); fabcasti@unina.it (F.C.); 2 Endoscopy Unit, Department of Gastroenterology, IRCCS Humanitas Research Hospital, 20089 Rozzano, Italy

**Keywords:** inflammatory bowel disease, IBD, nephropathies, IgAN, gut microbiota, gut-kidney axis

## Abstract

Inflammatory bowel disease (IBD) can extend beyond the gastrointestinal tract, affecting extraintestinal organs and significantly increasing morbidity and mortality. Despite early studies revealing kidney involvement in nearly a quarter of patients with IBD, renal manifestations have been notably overlooked. Among these manifestations, nephrolithiasis, obstructive uropathy, and fistula formation between the bowel and urinary tract are the most reported occurrences. Additionally, renal parenchymal involvement in IBD, including glomerulonephritis (GN), tubulointerstitial nephritis, and amyloidosis, has been documented. GN is particularly noteworthy, as a significant proportion of patients progress to end-stage kidney disease (ESKD). Although GN has long been recognized as a potential extraintestinal manifestation (EIM) of IBD, it has often been dismissed as an anecdotal association. Recently, several studies highlighted the clinical correlation between GN and IBD, suggesting a pathogenic interplay involving gut inflammation, dysbiosis, and intrinsic glomerular processes. Thus, our objective is to elucidate the basis of IBD-related nephropathies, with a specific focus on IgA nephropathy (IgAN) and the gut–kidney axis.

## 1. Introduction

Inflammatory bowel disease (IBD) can extend to other organs, significantly impacting health outcomes [1]. Extraintestinal manifestations (EIMs) are characterized as inflammatory conditions occurring in every system of the body and can broadly be classified as classical (inflammatory process occurring at distant sites), associations (associations with other immune-mediated disorders), and complications (complications of systemic inflammation); moreover, some manifestations occur as a side effect of IBD therapy [2,3]. These manifestations either result from the spread of immune responses from the intestine, which may occur independently even if they are influenced by IBD, or share similar environmental or genetic factors [4,5,6,7].

The renal manifestations associated with IBD have been relatively understudied despite early findings indicating kidney involvement in nearly a quarter of IBD patients. Initial research identified nephrolithiasis, obstructive uropathy, and fistula formation between the bowel and urinary tract as common occurrences [8,9]. Moreover, renal parenchymal involvement in IBD, including glomerulonephritis (GN), tubulointerstitial nephritis, and amyloidosis, has been documented. However, due to the absence of systematic analyses, the true prevalence remains unclear [10,11].

In recent years, the introduction of more potent medications for IBD treatment has heightened concerns about potential nephrotoxic effects, emphasizing the significance of this area of study [12]. Renal parenchymal involvement can affect various compartments, including the glomerular, tubular, or interstitial regions. GN is particularly noteworthy due to its significant implications, as approximately 20–30% of patients may progress to end-stage kidney disease (ESKD) if left untreated [13]. While GN has been acknowledged as a potential EIM of IBD for some time, it was frequently viewed as an anecdotal association [14]. Nevertheless, in recent years, several studies have emphasized the clinical correlation between GN and IBD, suggesting a pathogenic interplay involving gut inflammation, dysbiosis, and intrinsic glomerular processes [15,16,17].

Several studies have highlighted notable disparities in the gut microbiota (GM) composition between individuals with IBD and healthy controls [18,19]. Notably, patients with Crohn’s Disease (CD) often exhibit a decrease in the Firmicutes phylum, specifically *Faecalibacterium prausnitzii*, in their stool samples, while members of the Proteobacteria phylum, such as Enterobacteriaceae (including *Escherichia coli*), are frequently elevated compared to healthy controls [20,21,22]. This imbalance results in gut dysbiosis, characterized by a shift in the equilibrium between beneficial and potentially harmful microorganisms. An imbalance in microbial communities can disrupt the GM, potentially leading to intestinal dysbiosis by compromising the integrity of the intestinal barrier [23].

Furthermore, there are reports indicating that viable bacteria, primarily from the Proteobacteria phylum—particularly species such as *Escherichia coli*—can migrate from the gut to extraintestinal sites, including the kidneys. This bacterial translocation is often associated with bacterial dysbiosis, overgrowth, and weakened host immune defenses [24,25,26]. The pathological relationship between GM and kidney diseases is referred to as the gut–kidney axis, and it appears to play a role in various clinical manifestations, including IgA nephropathy (IgAN) [27,28].

In this narrative review, our objective is to elucidate the basis of IBD-related nephropathies, with a specific focus on IgAN and the gut–kidney axis.

## 2. The Gut–Kidney Axis

The connection between GM and chronic kidney disease (CKD) is termed the “gut–kidney axis” [29,30]. It is widely recognized that the intestinal microbiome, consisting of diverse microorganisms like bacteria, viruses, protozoa, and fungi, profoundly influences the host’s homeostasis during both health and disease [31,32]. Gut microbes and their metabolites play vital roles in maintaining gut epithelial integrity, providing energy, defending against pathogens, and modulating the immune system. Consequently, changes in microbial composition, known as dysbiosis, are thought to initiate the progression of several diseases, including CKD [33,34].

Emerging evidence indicates that GM contributes to CKD pathogenesis, interacting with systemic metabolic and inflammatory pathways [35,36]. Indeed, certain bacteria within the GM are responsible for synthesizing uremic toxins, which are implicated in CKD progression. Three bacterial families, namely Clostridiaceae, Enterobacteriaceae, and Verrucomicrobiaceae, harbor the tryptophanase gene responsible for indole production. Additionally, five families—Cellulomonadaceae, Dermabacteraceae, Micrococcaceae, Polyangiaceae, and Xanthomonadaceae—possess the uricase gene, while two families—Clostriadiaceae and Enterobacteriaceae—are capable of deaminating tyrosine into p-cresol [37]. These bacterial families are predominant in patients with ESKD. The high abundance of bacterial families possessing urease, uricase, and indole- and p-cresol-forming enzymes may hasten CKD progression by influencing the synthesis of uremic toxins [38]. Imbalances in the GM can lead to the heightened production of potentially harmful metabolites like indoxyl sulfate and p-cresyl sulfate, alongside increased permeability, and harm to the intestinal barrier. This damage to the barrier allows for greater exposure to uremic toxins and endotoxins [39]. The buildup of uremic toxins in the system exacerbates oxidative stress and inflammation, thereby fostering the onset of CKD [40].

In a recent systematic review, Stanford et al. highlighted that individuals with CKD demonstrate a decreased presence of the Prevotellaceae family and Roseburia genus, alongside an increase in potential pathobionts from the Enterobacteriaceae and Streptococcaceae families, as well as the *Enterococcus* genus, in comparison to healthy controls [41]. Additionally, considering kidney stones’ role in CKD development and progression, they also explored the correlation between GM and nephrolithiasis, reporting that this condition is associated with a significant reduction in *Bifidobacterium* and *Faecalibacterium* taxa, while *Bacteroides* was more prevalent [41].

Ren et al. investigated variations in microbial structure across different stages of CKD [42]. Their analysis, using linear discriminant analysis (LDA), revealed that Tenericutes and Mollicutes were elevated in CKD stages 1–2, Parasutterella was enriched in CKD stages 3–4, and Akkermansia, Blautia, and Verrucomicrobia were increased in CKD stage 5 [42]. Additionally, adults undergoing hemodialysis or peritoneal dialysis exhibited higher levels of Alphaproteobacteria, Streptococcaceae, and Streptococcus compared to controls [41].

**Figure 1:**
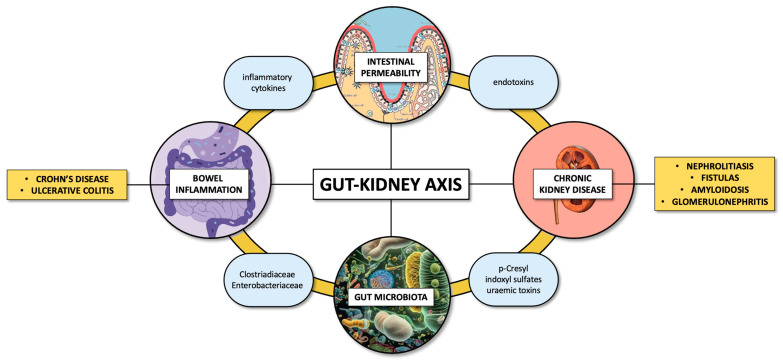
The gut–kidney axis: the link between IBD and CKD.

Figure 1 shows the key players in the gut–kidney axis.

## 3. Renal Involvement in IBD

Single or multiple EIMs may manifest before or after the onset of intestinal symptoms or the formal diagnosis of IBD. These manifestations include systemic inflammatory, autoimmunity susceptibility, and metabolic and nutritional dysregulation. The appearance of EIMs in patients with IBD is reported with variable frequency (from 4 to 49%) based on variability in the definition of EIMs, the heterogeneity of sites affected by inflammation, and the potential simultaneous involvement of multiple sites [5]. While almost any organ can be impacted, the joints, skin, eyes, liver, and biliary tract are most frequently mentioned as sites of EIMs.

Surprisingly, kidney involvement, reported in early studies in nearly 25% of IBD patients, has received considerably less attention [8]. The most prevalent occurrences included nephrolithiasis, obstructive uropathy, the development of fistulas connecting the bowel and urinary tract, and renal parenchymal involvement in forms such as glomerulonephritis, tubulointerstitial nephritis, and amyloidoisis. Furthermore, the spread of advanced drugs for treating IBD, some associated with nephrotoxicity, may impact the incidence of renal involvement. Approximately 30–50% of patients with ulcerative colitis and around 15% of those with Crohn’s disease are treated with 5-aminosalicylate (5-ASA), while anti-Tumor Necrosis Factor α (TNF-α) is prescribed to roughly 20–40% of IBD patients, especially those with moderate to severe disease. Both treatments have been associated with the development of progressive renal impairment.

### 3.1. Nephrolitiasis

Numerous studies demonstrate a heightened risk of nephrolithiasis in individuals with IBD compared to the general population [43]. The primary mechanisms leading to the formation of uric acid and calcium oxalate stones, two predominant types in IBD patients, involve diarrhea, malabsorption-related low urine volume and pH, and increased intestinal oxalate absorption-related hyperoxaluria. Additional mechanisms may contribute to the formation of oxalate stones. The bacterium *Oxalobacter formigenes* plays a role in degrading dietary oxalate, and its depletion in the gut can result in the increased absorption of oxalate. Administering *Oxalobacter* orally has been shown to reduce urinary oxalate concentration [44]. Low levels of antilithogenic substances such as magnesium and citrate in the urine also contribute to the formation of kidney stones in individuals with IBD [45]. Ideally, magnesium and citrate replacements should focus on normalizing urinary levels rather than serum levels [45]. Preventive measures include adopting a diet low in oxalate and fat, along with pyridoxine supplementation [46]. Oral cholestyramine, while increasing oxalate excretion, concurrently decreases citrate excretion [47]. The toxic effects of oxalate on renal epithelial and tubular cells can lead to oxalate nephropathy, characterized by persistent hyperoxaluria, and, in conjunction with stone formation, represents a significant but rare contributor to the development of CKD.

### 3.2. Fistulas

Crohn’s disease (CD), affecting the entire thickness of the intestinal wall, increases the susceptibility to bowel perforation and the development of fistulas [48,49,50]. The adhesion of inflamed intestinal segments to the bladder wall can lead to erosion and the development of colo- and entero-vesical fistulas in 2% to 4% of individuals with CD [51]. The appearance of fistulas may be preceded by subacute small bowel obstruction, especially in the presence of coexisting intestinal strictures [52]. Entero-vesical fistulas often coincide with the development of intrapelvic abscesses. Clinical symptoms of entero-vesical fistulas may include air bubbles in urine (pneumaturia), urinary tract infections, and the presence of stool in urine (fecaluria) [53]. Diagnosing this condition is often difficult, necessitating a high level of clinical suspicion. Cases might present with non-specific symptoms like fever and abdominal discomfort, lacking typical signs. Early and collaborative consultations involving specialists—such as a gastroenterologist, surgeon, radiologist, and pathologist—are essential to devise an effective, personalized treatment strategy. Intestinal Ultrasound (IUS) has emerged as a valuable, non-invasive tool in detecting colo-vesical fistulas, with the addition of an oral contrast potentially improving diagnostic accuracy [54]. Contrast-enhanced CT scans, widely used in clinical settings, offer a rapid and sensitive method for identifying fistulas, though they expose patients to radiation, an important consideration for young IBD patients who require frequent imaging over time [55]. Alternatively, MRI provides a precise visualization of fistula tracts without radiation exposure, offering a superior soft tissue contrast to CT while maintaining high sensitivity and specificity. Comprehensive imaging, often using multiple modalities, is usually necessary, and management typically integrates both medical and surgical approaches.

### 3.3. Amyloidosis

Serum amyloid protein (SAA) amyloidosis, also known as secondary amyloidosis, is an uncommon complication of IBD. It is caused by an extracellular deposition of insoluble amyloid fibrils derived from circulating acute-phase reactant serum amyloid A protein, with renal involvement recognized as the most frequently fatal manifestation of IBD-associated amyloidosis [56]. SAA amyloidosis typically emerges relatively late in the natural course of IBD due to prolonged and chronic uncontrolled inflammation, and its renal involvement typically presents with a nephrotic syndrome [4]. With a diagnostic gold standard represented by the kidney biopsy, SAA amyloidosis is associated with a faster progression to an ESKD, leading to increased rates of infection, severe sepsis, and multi-organ system involvement, and thus to reduced patient survival [57]. For these reasons, patients with IBD should be routinely screened for proteinuria with urinary dipsticks and serial measurement of SAA concentration. However, in about 15% of cases, neither proteinuria nor elevated serum creatinine is found [56]. Since SAA amyloidosis is strictly related to prolonged and uncontrolled inflammation, treatment should be targeted to the underlying IBD activity to reduce the new deposition of SAA.

### 3.4. Glomerulonephritis

Glomerulonephritis is another uncommon complication of IBD. Although infrequent reports have brought into question whether there is a causal link, the onset of glomerulonephritis often coincides with an acute exacerbation of intestinal inflammation, with renal function improving through treatment of the underlying IBD. Various types of glomerulonephritis associated with IBD have been documented, encompassing IgAN, crescentic glomerulonephritis, focal segmental glomerulosclerosis, membranous glomerulonephritis, minimal change disease, membranoproliferative glomerulonephritis, and mesangiocapillary glomerulonephritis [58]. In a histopathological examination reviewing kidney biopsy results from 83 IBD patients (45 with CD; 38 with UC), IgAN emerged as the most prevalent diagnosis [58].

### 3.5. Nephrotoxicity

#### 3.5.1. 5-ASA

The occurrence of nephrotoxicity linked to 5-ASA in IBD patients is uncommon and presents in an idiosyncratic manner, irrespective of the dosage, posing a challenge in establishing a definitive causal relationship explaining the numerous contradictory series reported in the literature. A genome-wide association study has established an association between human leukocyte antigen (HLA), particularly HLA-DRB1*03:01, and nephrotoxicity induced by 5-ASA [59]. Nephrotoxicity induced by 5-ASA typically occurs after a median treatment duration of three years, is more prevalent in males, and can manifest at any age. The most frequent histological observation is chronic tubulointerstitial nephritis. While uncommon, it is advisable to conduct an annual monitoring of renal function to identify nephrotoxicity associated with 5-ASA [12].

#### 3.5.2. Immunosuppressants

Tacrolimus and ciclosporin, both calcineurin inhibitors, are widely utilized in medical practice, particularly to prevent rejection incidents following solid-organ transplants [60]. Ciclosporin, in addition, has a recognized application in managing acute severe UC. However, its use is limited by its high toxicity profile, particularly nephrotoxicity, which affects its overall safety [12,61]. The nephrotoxic effects associated with ciclosporin are believed to stem from vasoconstriction of the kidney’s afferent arterioles, resulting in a reduced renal blood flow and lowered glomerular filtration rates [62]. Over time, this vasoconstriction may lead to chronic kidney damage, manifesting as interstitial fibrosis and a structural disruption of the arterioles [63].

#### 3.5.3. Biologic Drugs

TNF-α inhibitors, such as infliximab, adalimumab, certolizumab pegol, and golimumab, are increasingly being used in the treatment of Crohn’s Disease (CD) and Ulcerative Colitis (UC). There have been cases of infliximab-induced focal segmental glomerulosclerosis, which led to severe nephrotic syndrome in a UC patient [64]. The rise in novel therapeutic options for inflammatory bowel disease (IBD) has highlighted concerns regarding their toxicity and safety profiles. Vedolizumab (VDZ), a humanized monoclonal antibody targeting the α4β7 integrin, is prescribed for moderate to severe cases of UC and CD. A case report associated VDZ with acute tubulointerstitial nephritis; however, renal function was fully restored with glucocorticoid treatment, and the re-administration of VDZ was well-tolerated [65]. Ustekinumab (USK), an anti-IL-23 biologic, is another treatment option for moderate to severe UC and CD. While there has been an isolated case linking it to nephrotic syndrome secondary to focal segmental glomerulosclerosis [66], recent real-world studies confirm its safety and efficacy as a long-term treatment, with no renal complications reported [67]. Tofacitinib (TOFA), the first oral Janus kinase (JAK) inhibitor approved for UC treatment, has previously been linked to an increase in serum creatinine, though its clinical impact remains unclear [68]. Other JAK inhibitors, like upadacitinib (UPA) and filgotinib (FILGO), show no nephrotoxic effects [69]; however, pharmacokinetic studies indicate that FILGO concentration increases in patients with an estimated glomerular filtration rate (eGFR) below 60 mL/min/1.73 m^2^, prompting recommendations of dosage adjustments in such cases [70].

### 3.6. Dietary Factors

In patients with both IBD and CKD, dietary factors, such as oxalates, vitamin C, and caffeine, play a crucial role in disease management, influencing both gut and kidney health through a complex interplay of absorption, metabolism, and excretion.

The altered intestinal environment seen in IBD patients often leads to increased intestinal permeability and malabsorption, particularly of fats and bile acids. This malabsorption contributes to hyperoxaluria, where excess oxalate—derived from dietary sources or produced endogenously—accumulates in the body, significantly increasing the risk of calcium oxalate kidney stones [71]. In CKD, the role of oxalates becomes even more problematic. In CKD, gut dysbiosis can contribute to alterations in the metabolism of dietary components like oxalates. In this context, reduced *Oxalobacter formigenes* levels may impair oxalate degradation, potentially exacerbating calcium oxalate stone formation and renal damage [72].

Moreover, high doses of vitamin C—commonly used for its antioxidant properties—can exacerbate this condition because it metabolizes into oxalate, contributing further to the risk of oxalate nephropathy, particularly in CKD patients [73]. Therefore, while vitamin C is generally beneficial, excessive supplementation needs to be carefully monitored in these populations.

Caffeine consumption, often overlooked, also presents risks for both IBD and CKD patients. Caffeine has diuretic effects that can lead to dehydration, which increases urine concentration and enhances the risk of stone formation. Furthermore, caffeine increases calcium excretion in the urine, further compounding the risk of calcium oxalate stone formation in susceptible individuals [74]. While moderate caffeine intake is generally considered safe, in patients with IBD and CKD, particularly those prone to nephrolithiasis, careful regulation of caffeine consumption is advised.

Overall, managing the dietary intake of oxalates, vitamin C, and caffeine is essential in reducing the risk of complications such as kidney stones and further renal impairment in patients with comorbid IBD and CKD. Proper dietary interventions, including the restriction of high-oxalate foods and the cautious use of vitamin C supplements, alongside moderation of caffeine intake, are important strategies to minimize these risks and improve overall patient outcomes.

## 4. IgAN and IBD: Causation or Correlation?

IgAN is the most common primary glomerulonephritis, and it is characterized by a highly variable clinical presentation, ranging from microscopic haematuria to rapidly progressive glomerulonephritis and ESKD requiring chronic haemodialysis or kidney transplantation. The widely accepted pathogenesis of IgAN is the “multiple-hit hypothesis”. Gut dysbiosis can compromise the integrity of the intestinal mucosal barrier, leading to the release of galactose-deficient IgA1 (Gd-IgA1) into the bloodstream [75]. Upon binding with specific antibodies, the IgA1 deposits in renal tissues, contributing to the development of IgAN [75]. In this process, gut dysbiosis plays a role as a potential “trigger point”, being associated with increased gut permeability and aberrant immune responses, which may exacerbate kidney damage [75].

A recent study using metagenomic sequencing explored the characteristics and functional pathways of GM in patients with IgAN [76]. The findings revealed significant differences between IgAN patients and healthy controls. In the IgAN group, *E. coli* was notably more abundant compared to the control group (*p* < 0.05). Among patients with abnormal renal function, distinct microorganisms such as Enterococcaceae, *Moraxella*, and *Acinetobacter* were identified [76]. The study also demonstrated that the functional pathways of the gut microbiota that differed between IgAN patients and controls were primarily associated with bile acid metabolism [76].

Gleeson et al. explored how the GM, particularly *A. muciniphila* (a mucin-degrading bacterium), contributes to IgAN [77]. In humans and mice, *A. muciniphila* deglycosylated human IgA1, enabling it to travel from the gut to kidney glomeruli via circulation, causing autoimmune kidney disease [77]. Mice with human IgA1 and Fc α receptor I developed IgA nephropathy when colonized by *A. muciniphila*. In humans, the protective correlation between α-defensin 6 and *A. muciniphila* was lost in IgA nephropathy patients. Additionally, *A. muciniphila*-modified IgA1 was recognized by autoantibodies in patient sera, highlighting its role in disease pathogenesis [77].

A kidney biopsy series established the association between IgAN and IBD [78]. Therefore, there is a need to precisely understand the pathophysiological mechanisms underlying the various phenotypical manifestations to improve clinical and therapeutic management. More recently, some studies have shed light on the possible role of the gut–kidney axis in IgAN pathogenesis, with particular attention being paid to IBD. Patients with IBD showed IgAN as the most frequent abnormal finding upon kidney biopsy (24%), with a significantly higher prevalence compared to patients without IBD (8%, *p* < 0.001), which may reflect a common pathogenic mechanism between IgAN and IBD. This was also supported by a Swedish population-based cohort study, which demonstrated that patients with IgAN have a higher risk of developing IBD and that IgAN in IBD patients is more likely to progress to ESKD than in patients with isolated IgAN [79].

IgAN is considered as an immune-mediated disease influenced by a combination of genetic and environmental factors. The typical pathological feature is the development of immune complexes of galactose-deficient IgA1 (Gd-IgA1) which cannot be cleared from the circulation and thus deposit in the glomerular mesangium, potentially activating the complement system and triggering inflammation [80]. The development of immune complexes is found to be a heritable trait [81] and genome-wide association study (GWAS) data sets have identified multiple susceptibility loci for IgAN along with several risk alleles associated with intestinal epithelial barrier maintenance and mucosal immunity [82,83,84]. Some of the loci involved in IgAN, such as CARD9 or HORMAD2, are shared with IBD [85,86], and have also been associated with immune-mediated diseases, such as Systemic Lupus Erythematosus (SLE) [87].

Two specific single-nucleotide polymorphisms (SNPs), namely rs4151657 and rs549182, have been linked to IgAN [88]. The SNP rs4151657 is found within the intron of the complement factor B (CFB) gene, which is crucial for initiating the alternative complement pathway, thought to be a primary mechanism in IgAN development. Studies suggest that CFB is locally elevated in colonic biopsies from patients with IBD [89,90,91]. Additionally, these patients exhibit signs of complementary activation within the intestinal mucosa and increased circulating complement factors. This finding supports the idea that alternative pathway activation in IBD, coupled with the release of complement components and inflammatory mediators into the bloodstream, may contribute to IgAN. Abnormalities in IgA, such as irregular glycosylation, self-aggregation, receptor shedding, or the formation of immune complexes (ICs) with dietary antigens, are known to trigger inflammatory conditions impacting both the intestines and kidneys. Furthermore, LIGHT, an essential molecule for the activation of T cells that migrate to the gut, may provoke intense gut inflammation and IgA deposits in the kidneys, potentially contributing to IBD, IgAN, and even celiac disease [92,93].

On the other hand, the SNP rs549182 is mapped to the NOTCH4 gene and was found to be correlated to the process of glomerulosclerosis, which occurs in IgAN [94]. Since it is involved in cell proliferation, inflammation, and innate immunity, the Notch receptor family and signaling pathways are also involved in the development of IBD. Moreover, in patients with IgAN, advanced glomerulosclerosis and tubulointerstitial changes were found to be more severe in patients with IBD than in patients without IBD [95], leading to the hypothesis that IBD can promote inflammation in IgAN. In fact, patients with IBD are more exposed to nephrotoxicity due to the risk of dehydration, which can activate the renin–angiotensin–aldosterone system involved in glomerular ischemia and interstitial fibrosis, and due to the systemic inflammation characterized by the production of IL-23 and TNF-α, which could contribute to the exacerbation of tubulointerstitial lesions in IgAN. Additionally, it has been reported that CD16+ macrophages may contribute to intestinal fibrosis [96], as well as abnormal B cell activation [97], which can also play a role in interstitial inflammation in CKD, including IgAN [98]. Recently, it was found that IBD and IgAN share some changes in SDEGs, miRNAs and SIRGs, which are involved in the T cell receptor (TCR) pathway, related to humoral immunity, leading to the activation and differentiation of CD4+ T cells. Moreover, it was found that these SDEGs and SIRGs were related to the differentiation of Th17 cells, which cause autoimmunity and inflammation, promote the immune response, and activate various inflammatory pathways through the IL-17 signaling pathway. Therefore, infections and other factors can up-regulate the expression of Th17 cells and Th17-related cytokines in mucosal immunity, inducing or aggravating IgAN and IBD. This may represent not only a common pathogenetic mechanism but also a potential therapeutic target [99]. Moreover, SDEGs and SIRGs are both associated with the regulation of inflammatory mediators of transient receptor potential (TRP) channels. These channels are distributed throughout the gastrointestinal tract and play a role in intestinal inflammation. TRP channels are expressed in immune cells and contribute to the immune response by activating macrophages and CD4+T cells. Several studies have implicated them in the immune pathogenesis of IBD [100,101]. Therefore, the modulation of TRP channels, either through inhibition or activation, could be a therapeutic strategy for intestinal inflammation [99]. Additionally, since inhibiting TRP channel-related genes, such as TRPC5, could protect podocytes in the kidney and prevent kidney failure, TRP channels may also emerge as promising targets for the prevention and treatment of both IgAN and IBD [102].

Table 1 shows the main studies that explored the relationship between IBD and IgAN [16,17,101,103,104].

## 5. Conclusions

Renal involvement in IBD is often underestimated, despite its affecting nearly a quarter of patients. Emerging research highlights the gut–kidney axis as an influential factor in nephropathy development, particularly through its impact on systemic inflammation and metabolite homeostasis. Dysbiosis-induced systemic inflammation may exacerbate renal involvement in IBD, although the causality of this remains under investigation. Notably, IgAN stands out as an immune-mediated condition influenced by both genetic and environmental factors. Studies indicate that patients with IgAN have a higher risk of developing IBD, and when IgAN co-occurs with IBD, there is an increased likelihood of progression to ESKD, suggesting a pathogenic link between these conditions through the gut–kidney axis. Among IBD treatments, certain drugs, such as 5-ASA and anti-TNF-α agents, may increase the risk of renal complications, whereas other biologics and small molecules show minimal nephrotoxic effects. Thus, the careful selection and monitoring of therapies are essential to mitigate potential renal risks in IBD patients. Future research should focus on understanding the detailed mechanisms of the gut–kidney axis and developing targeted therapies to improve the outcomes of patients with IBD and nephropathies.

## Figures and Tables

**Table 1:** Main studies that explored the relationship between IBD and IgAN.

Author, Year	Study Design	Study Population	Intervention	Outcomes	Key Findings
Xiao et al., 2022 [103]	Mendelian randomization (MR) study	The summary statistics of exposures included IBD (N = 31,665 cases and 33,977 controls), UC (N = 6,968 cases and 20,464 controls), and CD (N = 5956 cases and 14,927 controls). For IgAN, the GWAS summary statistics included 977 subjects with IgAN and 4980 disease-free controls	In this two-sample MR study, significant and independent single-nucleotide polymorphisms (SNPs) were chosen as IVs to clarify the causal association at the genetic level using the GWAS data	Clarify if IBD, UC, and CD have a potential causal effect on IgAN and whether a reverse causal effect exists	The results of this two-sample MR analysis suggested that IBD, UC, and CD were causally associated with the risk of IgAN, while there was no sufficient evidence for the causal effect of IgAN on IBD, UC, or CD
Qing et al., 2022 [101]	Case-control study	45UC patients (adults), 47CD patients (adults) and 47 healthy controls (adults). 12IgAN patients and 8 healthy controls	The differential expression analysis and weighted gene co-expression network analysis (WGCNA) were conducted on gene expression data of IgAN, UC and CD, which were obtained from the Gene Expression Omnibus (GEO)	Focusing on the genetic effects and abnormal immunity in IgAN and IBD	This work revealed that the differentiation of Th17 cells may mediate the abnormal humoral immunity in IgAN and IBD patients and identified novel gene candidates that could be used as biomarkers or potential therapeutic targets
Nurmi et al., 2021 [104]	Retrospective study	629 patients with newly diagnosed IgAN during the years 1976–2012	Data on diagnosis of IBD and celiac disease were retrospectively collected from medical records. Further, to detect unrecognized celiac disease, IgA-class tissue transglutaminase antibodies (tTGA) were measured from serum samples taken at the time of kidney biopsy during years 1980–2012 (defined as screen-detected celiac disease autoimmunity)	Studying the prevalence of IBD and celiac disease in IgAN patients over time	The prevalence of IBD increased over time in IgAN patients, which exceeds the prevalence of 0.6% in the Finnish general population
Lian et al., 2023 [16]	Mendelian randomization (MR) study	They selected a group of single-nucleotide polymorphisms (SNPs) specific to IBD as instrumental variables from a published genome-wide association study (GWAS) with 86,640 individuals of European ancestry	Summary statistics for multiple kidney diseases were obtained from the publicly available GWAS. Genetic data from one GWAS involving 210 extensive T-cell traits were used to estimate the mediating effect on specific kidney disease	Examining the causal association between genetically predicted IBD and the risk of multiple kidney diseases. Evaluating which type of kidney disease primarily reflects this causal relationship	This study provides genetic evidence supporting a positive causal association between IBD, including its subclassification as ulcerative colitis and Crohn’s disease, and the risk of IgAN. However, no causal association was found between IBD and other types of kidney diseases
Joher et al., 2021 [17]	Retrospective multicentre study	24 patients with biopsy-proven IgAN occurring in a context of CD or UC	These patients were compared with 134 patients with primary biopsy-proven IgAN and 19 patients with IBD-associated IgAN identified through a systematic literature review	Assessing the clinical significance of the association between IgAN and IBD by reviewing clinical, histological and therapeutic data and outcomes for patients with IgAN occurring in a context of CD or UC	IBD-associated IgAN has frequent inflammatory lesions at onset and variable long-term outcomes. Interestingly, the IgAN diagnosis was not associated with IBD flare. TNF-α blockade had no impact on the presentation and course of IBD-IgAN

## Data Availability

Not applicable.
